# Supplementary material for: Shorter Chitin Nanofibrils Enhance Pickering Emulsion Stability: Role of Length and Interfacial Network
Source: Foods. 2025 Dec 26;15(1):76. doi: 10.3390/foods15010076 (PMC12785728; doi:10.3390/foods15010076)
Supplement: Supplementary file 1 [file foods-15-00076-s001.zip › foods-4036560-supplementary.pdf]

Supporting Information for

Shorter Chitin Nanofibrils Enhance Pickering Emulsion Stability: Role of Length and Interfacial Network

Qiyue Yang [1,2], Congying Chen [1,2], Xiaoyi Luo [2], Ruoxin Li [2], Zhenjun Zhu [3], Yehui Zhang [2], Xinglong Xiao [1,*] and Wenjuan Jiao [2,*]

1 School of Food Science and Engineering, South China University of Technology, Guangzhou 510640, China; yqy51522@163.com (Q.Y.); c1030514976@163.com (C.C.)

2 Key Laboratory of Functional Foods, Ministry of Agriculture and Rural Affairs, Guangdong Key Laboratory of Agricultural Products Processing, Sericulture & Agri-Food Research Institute Guangdong Academy of Agricultural Sciences, Guangzhou 510610, China; 18739896783@163.com (X.L.); 13131171362@163.com (R.L.); zhangyehui@gdaas.cn (Y.Z.)

3 Department of Food Science and Engineering, College of Life Science and Technology, Jinan University, Guangzhou 510632, China; zzj1904@jnu.edu.cn

* Correspondence: fexxl@scut.edu.cn (X.X.); jiaowenjuan@gdaas.cn (W.J.); Tel.: +86-13826279058 (X.X.); +86-13710154267 (W.J.)

This Supporting Information document contains one (1) figure and two tables (2) in three (3) pages.

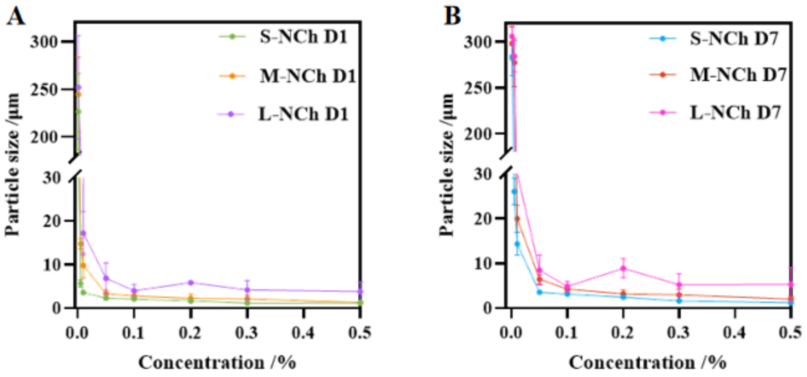

Figure S1. Particle size of Pickering emulsions stabilized by NCh with different length. (A) Day1, (B) Day7.

Table S1. Potential comparison of NCh stabilized Pickering emulsions with different length diameter ratios on Day1

Nanofibril concentration /%	Potential /mV L-NCh	M-NCh	S-NCh
0.5	59.4 ± 0.9899^{Bd}	63.9 ± 1.1898^{Ac}	65.4 ± 0.665^{Ab}
0.3	64.5 ± 0.4546^{Bbc}	69.8 ± 0.4497^{Ab}	70.2 ± 0.9393^{Aa}
0.2	62.7 ± 0.5354^{Cc}	72.6 ± 0.9463^{Aa}	69.7 ± 0.3859^{Ba}
0.1	68.5 ± 0.2494^{Ba}	69 ± 0.2625^{Bb}	70.7 ± 0.3859^{Aa}
0.05	66.1 ± 1.1225^{Ab}	67.1 ± 0.2449^{Ab}	41.7 ± 1.0614^{Bd}
0.01	57.9 ± 0.6342^{Bd}	59.7 ± 0.4243^{Ad}	42.4 ± 0.5907^{Cd}
0.005	43.4 ± 1.6269^{Be}	53.9 ± 1.4384^{Ae}	54.6 ± 0.17^{Ac}
0.001	16.3 ± 1.4704^{Bf}	24.6 ± 0.6848^{Af}	22.3 ± 1.7932^{Ae}

Letters A-C in the same row indicated significant differences ($P < 0.05$). Letters a-f in the same column indicated significant differences ($P<0.05$). Data are expressed as mean ± standard deviation (n = 3).

Table S2. Potential comparison of NCh stabilized Pickering emulsions with different length diameter ratios on Day7

Nanofibril concentration /%	Potential /mV L-NCh	M-NCh	S-NCh
0.5	62.4 ± 1.9799^{Ac}	63.7 ± 0.2625^{Ab}	64.9 ± 0.4497^{Ab}
0.3	62.4 ± 0.4243^{Cc}	67.9 ± 0.216^{Aa}	65.5 ± 0.7483^{Bb}
0.2	64.7 ± 0.1247^{Ab}	63.7 ± 0.7118^{Ab}	60.7 ± 0.5099^{Bc}
0.1	67.2 ± 0.9933^{Aa}	62.9 ± 0.6532^{Bb}	60.4 ± 0.9534^{Cc}
0.05	65.7 ± 0.5312^{Bab}	67.9 ± 0.6799^{Aa}	66 ± 0.6683^{Bb}
0.01	56.7 ± 0.8602^{Ce}	69.3 ± 0.5354^{Ba}	72.2 ± 1.6269^{Aa}
0.005	58.9 ± 1.3275^{Bd}	42.8 ± 3.4989^{Cc}	67.7 ± 2.6944^{Ab}
0.001	4.3 ± 0.4815^{Cf}	11.4 ± 2.7439^{Bd}	31.4 ± 3.7974^{Ad}

Letters A-C in the same row indicated significant differences (*P < 0.05*). Letters a-f in the same column indicated significant differences (*P<0.05*). Data are expressed as mean ± standard deviation (n = 3).
